# Inter‐Rater Reliability in Pre‐Anaesthetic Airway Assessment: A Protocol for a Systematic Review

**DOI:** 10.1111/aas.70354

**Published:** 2026-10-09

**Authors:** Constance Thyra Giertz Christiansen, Mathilde Nellemann Jensen, Moritz Kilian German Denneborg, Sofie Amalie Bosholdt, Janus Christian Jakobsen, Rasmus Tingkær Hesselfeldt, Morten Hylander Møller, Andreas Møgelmose, Markus Harboe Olsen, Michael Seltz Kristensen, Katrine Bredahl Buggeskov

**Affiliations:** ^1^ Department of Anaesthesia, Surgery and Trauma Centre ‐ Rigshospitalet Copenhagen University Hospital Copenhagen Denmark; ^2^ Copenhagen Trial Unit, Centre for Clinical Intervention Research ‐ Rigshospitalet Copenhagen University Hospital Copenhagen Denmark; ^3^ Department of Regional Health Research, The Faculty of Health Sciences University of Southern Denmark Odense Denmark; ^4^ Department of Intensive Care Copenhagen University Hospital ‐ Rigshospitalet Copenhagen Denmark; ^5^ Department of Clinical Medicine Copenhagen University Hospital Copenhagen Denmark; ^6^ Visual Analysis and Perception Lab Aalborg University Aalborg Denmark; ^7^ Department of Neuroanaesthesiology, The Neuroscience Centre – Rigshospitalet Copenhagen University Hospital Copenhagen Denmark

## Abstract

**Background:**

Pre‐anaesthetic airway assessment relies on subjective clinical judgement, whether performed through direct physical examination, such as Mallampati classification or thyromental distance, or through composite scoring systems that combine several such components, including the Simplified Airway Risk Index, the El‐Ganzouri Risk Index and the Wilson score. The clinical value of these assessments depends not only on their predictive accuracy but also on whether they can be applied consistently across assessors. However, evidence on the inter‐rater reliability, agreement or variability of these composite tools and their individual components remains scattered across the literature and has not previously been systematically synthesised. This systematic review therefore aims to identify, assess and synthesise the available literature on inter‐rater reliability, agreement or variability in composite airway assessment tools and individual airway assessment components in the context of pre‐anaesthetic or preoperative assessment. Based on previous inter‐observer studies of individual airway assessment components, we hypothesise that inter‐rater reliability of these tools and components will predominantly range from moderate to excellent.

**Methods:**

This systematic review protocol has been developed in accordance with Preferred Reporting Items for Systematic Review and Meta‐Analysis Protocols (PRISMA‐P) and informed by the PRISMA 2020 statement and the Cochrane Handbook for Systematic Reviews. A systematic search will be conducted in MEDLINE, Embase, CINAHL and CENTRAL, supplemented by reference list screening and forward citation tracking of foundational articles. Studies involving adult patients or healthy volunteers, in which two or more assessors independently evaluated a composite airway assessment tool or individual airway assessment component through physical assessment of a real participant, will be eligible for inclusion. Two reviewers will independently screen studies and extract data using a standardised, piloted form. Methodological quality will be assessed using a checklist adapted from the Guidelines for Reporting Reliability and Agreement Studies. Findings will be synthesised primarily narratively; quantitative synthesis may be considered where studies are judged sufficiently homogeneous.

**Conclusion:**

This systematic review will synthesise the available literature on inter‐rater reliability, agreement or variability in composite airway assessment tools and individual airway assessment components and may help clarify the consistency of these routinely used pre‐anaesthetic assessments across both clinical and research populations.

## 1 Introduction

### 1.1 Background

Pre‐anaesthetic assessment is a cornerstone of perioperative care. It is intended to identify patient‐related risk factors, anticipate challenges during anaesthesia and support decisions regarding planning and allocation of clinical resources before, during and after surgery [1, 2]. In this assessment, airway evaluation plays a particularly important role, as unanticipated difficult airway management can have serious consequences and often requires timely preparation, appropriate equipment and experienced personnel.

Airway assessment is commonly based on a combination of clinical bedside tests and observations, including Mallampati classification [3], mouth opening, thyromental distance, neck mobility, ability to prognath [4] and upper lip bite test [5]. These components may be interpreted individually or combined into composite airway assessment tools. Such tools include the Simplified Airway Risk Index (SARI) and the El‐Ganzouri Risk Index, which were introduced within the same original publication, with SARI representing a simplified, operational version of the underlying multivariable model [6], as well as the Wilson score [4], the LEMON criteria [7], the MACOCHA score [8], the Arné score [9] and the Naguib score [10]. Composite tools aim to provide a more structured assessment of difficult airway risk by combining several clinical predictors into a single score or risk estimate.

However, the value of any clinical assessment depends not only on its ability to predict difficult airway management, but also on whether it can be applied consistently between assessors. Many airway assessment components consist of subjective judgement, patient cooperation, correct positioning and consistent interpretation of anatomical findings. Even apparently simple measurements may vary depending on how the assessment is performed [11, 12]. Consequently, inter‐rater reliability, agreement and variability are clinically relevant: if different clinicians assign different scores to the same patient, the assessment may become less useful for perioperative planning and communication.

The clinical context of airway assessment is also evolving. The increasing use of video laryngoscopy has reduced the importance of some challenges associated with difficult direct laryngoscopy and may change the consequences of traditional airway predictors [13, 14]. Predicting difficult or failed video laryngoscopy and video‐laryngoscopy‐guided intubation is thus a newer challenge. Thus, structured airway assessment remains relevant. Video laryngoscopy does not eliminate all airway‐related risks, and clinicians still need to anticipate potential difficulties with mask ventilation, supraglottic airway placement, tube passage, rescue strategies or the need for senior assistance [15]. Reliable airway assessment may therefore continue to support preparation and risk communication, even in contemporary airway management practice.

A systematic review is needed to identify, assess and synthesise the literature on inter‐rater reliability, agreement and variability in composite airway assessment tools and individual airway assessment components. Such a review may clarify which assessments have been studied, how reliability has been quantified and whether current evidence supports the consistent use of these assessments in pre‐anaesthetic clinical decision‐making. Previous inter‐observer studies of individual airway assessment components have generally reported moderate‐to‐excellent reliability [11, 12, 16]. Based on these findings, we hypothesise that inter‐rater reliability of both composite airway assessment tools and individual components will predominantly fall within this moderate‐to‐excellent range.

### 1.2 Review Question and Objectives

Among patients or study participants undergoing pre‐anaesthetic airway assessment, what does the literature report on the inter‐rater reliability, agreement or variability of composite and individual‐component airway assessment tools?

The objective of this systematic review is to identify, assess and synthesise the available literature addressing the review question above, with specific attention to:

1. How inter‐rater reliability, agreement or variability has been quantified and reported across included studies
2. How reported estimates vary according to assessment method, assessor characteristics and population type (patients versus healthy volunteers or research‐setting participants); and
3. The methodological quality of the underlying evidence.

## 2 Methods

This systematic review protocol has been developed in accordance with the Preferred Reporting Items for Systematic Review and Meta‐Analysis Protocols (PRISMA‐P) [17] (Appendix 4) and informed by the PRISMA 2020 statement [18] (Appendix 5) and methodological guidance from the Cochrane Handbook for Systematic Reviews [19]. The review will be conducted to identify, assess and synthesise studies reporting inter‐rater reliability, agreement or variability in composite airway assessment tools and individual airway assessment components in the context of pre‐anaesthetic or preoperative assessment. The study selection process will be documented using a PRISMA flow diagram [18].

### 2.1 Eligibility Criteria

Studies will be eligible for inclusion based on the following criteria.

#### 2.1.1 Population and Setting

Adult participants will be eligible for inclusion, comprising both patients undergoing pre‐anaesthetic assessment and healthy volunteers or other study participants assessed using standardised airway assessment methods outside a clinical perioperative setting (e.g., in a research or training setting). Provided the airway assessment was performed on a real human participant rather than a manikin or simulator. Eligibility further requires that the airway assessment methods correspond to those used in, or directly transferable to, adult clinical pre‐anaesthetic practice.

Age eligibility will be based on how each study itself defines its population as adult (e.g., an explicit age threshold, inclusion criterion or population description). Where this is not stated, eligibility will instead be based on the reported age range or mean/median age of participants.

Studies focusing exclusively on paediatric airway assessment methods or tools will be excluded, as paediatric airway anatomy and assessment differ substantially from adult practice, and dedicated paediatric‐specific tools are typically used rather than the adult‐focused composite tools and components addressed in this review. Studies with mixed adult and paediatric populations will be eligible only if adult‐specific data can be extracted or reported separately; otherwise, they will be excluded. No restrictions will apply to sex, comorbidity burden or surgical specialty.

Studies conducted in clinical perioperative populations and studies conducted in healthy volunteers or research/training settings will be treated as analytically distinct subgroups throughout data synthesis, given that inter‐rater agreement observed under standardised research conditions may not directly generalise to clinical pre‐anaesthetic practice.

#### 2.1.2 Assessment Types

Studies will be included if they assess inter‐rater variability, agreement or reliability among two or more assessors for at least one of the following: individual airway assessment components or composite airway assessment tool (see Figure 1).

**FIGURE 1:**
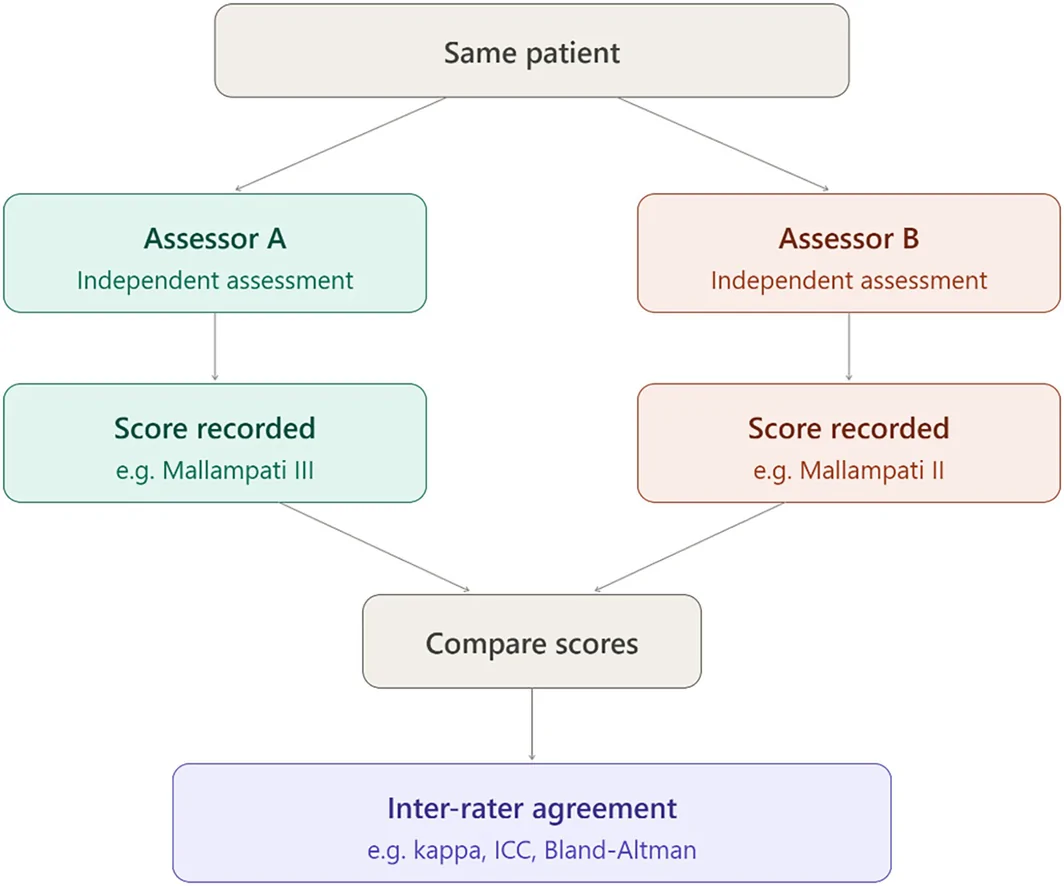
Illustration of the inter‐rater reliability concept. The same patient is independently assessed by two assessors (Assessor A and B), each producing a score (e.g., Mallampati classification). The two scores are then compared to calculate a statistical measure of agreement between assessors (e.g., Cohen's kappa, ICC or Bland–Altman analysis).

Individual airway assessment components may include, but are not limited to, Mallampati classification [3], mouth opening, thyromental distance, neck mobility, ability to prognath [4], upper lip bite test [5]. Composite airway assessment tools may include, but are not limited to, the Simplified Airway Risk Index (SARI) [20], the El‐Ganzouri Risk Index [6], the Wilson score [4], the LEMON criteria [7], the MACOCHA score [8], the Arné score [9], the Naguib score [10]. Inter‐rater reliability may be assessed using any statistical method, including Cohen's kappa [21], weighted kappa [22], Fleiss’ kappa [23], intraclass correlation coefficients [24], percentage agreement, Bland–Altman analysis (limits of agreement) [25], Gwet's AC1 (first‐order agreement coefficient) or AC2 [26], Spearman's or Pearson's correlation coefficient [27, 28], or other measures of agreement, variability, or reliability.

Studies reporting only intra‐rater (test–retest) reliability, without a comparison between two or more independent assessors, will be excluded. Studies reporting both inter‐rater and intra‐rater reliability will be eligible for inclusion, provided that inter‐rater estimates are reported separately or can be extracted independently of intra‐rater estimates; only the inter‐rater estimates will be extracted and synthesised. Studies in which inter‐rater and intra‐rater variance components cannot be statistically disentangled will be excluded.

Scores that quantify difficulty encountered during or after laryngoscopy or tracheal intubation itself (e.g., the Intubation Difficulty Scale [29] and the Videolaryngoscopic Intubation and Difficult Airway Classification (VIDIAC) score [30]) will not be considered pre‐anaesthetic airway assessment tools within the scope of this review, as these characterise intra‐ or post‐procedural findings rather than a pre‐anaesthetic clinical assessment.

#### 2.1.3 Study Design and Publication Format

Primary research studies published in peer‐reviewed journals will be eligible for inclusion. Studies involving a physical airway assessment of a real participant, whether a patient or a healthy volunteer, will be eligible, regardless of whether the assessment was performed in a clinical perioperative setting or a research/training setting. Studies based solely on surveys, written vignettes, hypothetical scenarios, or assessments performed on manikins or simulators, rather than on real participants, will be excluded, as these do not reflect inter‐rater variability in a physical clinical assessment. Protocols, case reports, opinion papers, editorials and conference abstracts without full‐text publications will be excluded. Letters and correspondence will be eligible for inclusion where they report original data from a reliability or agreement study; letters or correspondence that solely comment on, or discuss, previously published work without presenting original data will be excluded.

Only studies available as full‐text publications will be included. Full‐text peer‐reviewed publications will be required because detailed information on rater procedures, assessment conditions, participant selection, statistical methods and reliability estimates is necessary for eligibility assessment, data extraction and methodological quality appraisal.

#### 2.1.4 Language and Date Restrictions

No language or publication year restrictions will be applied during the search process. All records identified by the search will be considered for screening. Where potentially eligible studies are published in languages not spoken by the review team, translation will be sought or performed where feasible.

### 2.2 Outcomes

#### 2.2.1 Primary Outcomes

The primary outcome will be the inter‐rater reliability, agreement or variability of composite airway assessment tools used in pre‐anaesthetic or preoperative assessment.

Composite airway assessment tools may include, but are not limited to, the Simplified Airway Risk Index (SARI) [20], the El‐Ganzouri Risk Index [6], the Wilson score [4], the LEMON criteria [7], the MACOCHA score [8], the Arné score [9], the Naguib score [10] or other multicomponent airway assessment scores. Reliability, agreement, or variability estimates will be extracted as reported by the included studies and handled according to the approach described in the section on *reliability estimates and reporting modalities*.

#### 2.2.2 Secondary Outcomes

Secondary outcomes will include inter‐rater reliability, agreement or variability of individual airway assessment components. Individual airway assessment components may include, but are not limited to, Mallampati classification [3], mouth opening, thyromental distance, neck mobility, ability to prognath [4], upper lip bite test [5] or related airway measures. Reliability, agreement or variability estimates will be extracted as reported by the included studies and handled according to the approach described in the section on *reliability estimates and reporting modalities*.

#### 2.2.3 Additional Descriptive and Methodological Variables

To support interpretation of the primary and secondary outcomes, additional descriptive and methodological variables will be extracted, including clinical or research setting, participant population, assessor characteristics, timing of assessments and whether assessments were performed independently. These variables will not be considered outcomes but will be used to describe the included studies and explore sources of clinical or methodological heterogeneity in the synthesis.

Where reported, correlation coefficients (Spearman's or Pearson's [27, 28]) will also be extracted but will not be treated as measures of reliability or agreement, as correlation and agreement are distinct statistical concepts that can diverge substantially in practice [31, 32].

### 2.3 Information Sources

A systematic literature search will be conducted in the electronic databases MEDLINE (Ovid), Embase (Ovid), CINAHL (EBSCOhost) and the Cochrane Central Register of Controlled Trials (CENTRAL; Cochrane Library, Wiley). To ensure comprehensive coverage, additional studies will be identified through: (1) screening of reference lists from included articles and relevant reviews and (2) a forward citation search on selected foundational articles describing widely used airway assessment components, composite airway assessment tools, or precursor instruments, including Mallampati et al. [3]; Wilson et al. [4]; and El‐Ganzouri et al. [6], performed using Web of Science. Records identified through citation tracking will be screened using the same eligibility criteria as the database search results and reported separately in the PRISMA flow diagram [18]. Forward citation tracking will be performed after database screening is complete, using the finalised list of included studies to confirm that the foundational articles are appropriately represented, and to identify any additional eligible studies not captured by the database search.

The date of the final search will be reported in the final review, and a supplementary search will be conducted before data extraction to assess all potential articles.

### 2.4 Search Strategy

The search strategy will be developed in collaboration with an information specialist and will be designed to identify studies reporting inter‐rater reliability, agreement or variability in composite airway assessment tools or individual airway assessment components.

The search strategy will be structured around three main concepts combined using Boolean operators:

1. Composite airway assessment tools or individual airway assessment components
2. Pre‐anaesthetic or preoperative assessment: and
3. Inter‐rater variability, agreement or reliability

Each concept will include controlled vocabulary terms, such as MeSH and Emtree terms, and free‐text terms, including synonyms, spelling variations and relevant abbreviations. The concepts and corresponding search terms will be presented in Appendix 1. The search strategy will be adapted to the syntax and indexing system of each included database.

Before execution of the final search, the complete search strategy will be reviewed and refined iteratively in collaboration with the information specialist. It will also be reviewed by the co‐author group, including authors with clinical expertise in anaesthesia and airway management, to support conceptual completeness of the included composite airway assessment tools and individual airway assessment components.

### 2.5 Study Selection Process

All identified records from the database search will be imported into a reference management software, and duplicates will be removed prior to screening. Study selection will be conducted using Covidence (Veritas Health Innovation, Melbourne, Australia). Two reviewers will independently screen titles and abstracts against the eligibility criteria. Studies considered potentially eligible by either reviewer will proceed to full‐text assessment.

Records identified through reference list screening and forward citation tracking will be screened separately, using the same eligibility criteria and process as described above.

Full‐text articles will subsequently be assessed independently by two reviewers. Reasons for exclusion at the full‐text stage will be documented. Disagreements at any stage of the study selection process will be resolved through discussion and, if necessary, consultation with a third reviewer. The study selection process will be documented using a PRISMA flow diagram [18], distinguishing records identified via database searching from records identified via other methods and including the number of records identified, screened, assessed for eligibility, excluded and included in the final review. Full‐text articles that cannot be retrieved after reasonable attempts will be recorded as unavailable and excluded, with this reason documented in the PRISMA flow diagram.

### 2.6 Data Extraction

A standardised data extraction form will be developed based on the review objectives and eligibility criteria. The form will be piloted independently by both reviewers on a purposive sample of known eligible studies and refined as necessary based on the results of piloting. The finalised form will subsequently be applied to all studies identified as eligible through the full systematic search.

Where a single study is reported across multiple publications, these will be treated as one study and data will be extracted from the publication reporting the most complete relevant information, supplemented by data from companion publications where necessary.

Data extraction will be performed independently by two reviewers. Where relevant data are missing, unclear, or cannot be determined from the published report, study authors will be contacted for clarification or additional data. If no response is received, or if requested data cannot be obtained, the relevant item will be recorded as not reported. Discrepancies between reviewers will be resolved through discussion and, if necessary, consultation with a third reviewer.

### 2.7 Data Items

The following data will be extracted from each included study:

Study characteristics

- Author(s)
- Year of publication
- Country
- Study design
- Study setting

Population and setting

- Population type (patients undergoing pre‐anaesthetic/preoperative assessment vs. healthy volunteers or other study participants)
- Number of participants
- Where reported, the distribution of airway risk characteristics within the sample
- Surgical specialty or clinical context, where applicable
- Setting of the airway assessment (clinical perioperative setting vs. research/training setting)

Assessment type

- Type of assessment evaluated: composite airway assessment tool or individual airway assessment component
- Name of composite airway assessment tool, where applicable and its component variables (cross‐referenced with the classification in Appendix 2; new tools not covered in Appendix 2 will be added)
- Individual airway component(s) evaluated, where applicable
- Scoring categories or score range used

Assessment characteristics

- Number and type of assessors
- Assessor profession or level of experience
- Number of raters included in each reliability estimate
- Whether the same raters assessed all participants
- Timing of assessments
- Independence and blinding of assessments (i.e., whether ratings were performed independently, and assessors were unaware of each other's ratings)

Inter‐rater variability

- Method used to assess reliability or agreement
- Exact statistical model or type used (e.g., two‐way random/fixed effects, single‐measures/average‐measures ICC [24]; unweighted/weighted kappa [21, 22] and weighting scheme), where reported
- Number of participants or ratings included in the reliability analysis, where this differs from the total study sample (e.g., due to missing data)
- Prevalence or category distribution of ratings, where reported (for categorical outcomes)
- Reported reliability or agreement estimate
- Confidence intervals
- Author's interpretation of reliability or agreement
- Where a study reports multiple inter‐rater estimates for the same assessment tool or component (e.g., across multiple assessor pairs, assessor groups, clinical settings, populations, or statistical measures), each estimate will be extracted separately, together with the corresponding population, assessor group, setting and statistical measure to which it relates

If relevant data items are not reported or cannot be determined from the included study, they will be recorded as not reported.

### 2.8 Methodological Quality Assessment

Because no single established tool fully addresses the range of reliability and agreement studies expected in this review, methodological quality will be assessed independently by two reviewers using an adapted checklist informed by the Guidelines for Reporting Reliability and Agreement Studies (GRRAS) [33, 34], supplemented with domains considered directly relevant to methodological quality in inter‐rater studies. GRRAS was selected as the primary reference framework because it was developed specifically for studies reporting reliability, agreement, or measurement variability between raters, in contrast to measurement‐property frameworks developed primarily for multi‐item self‐report instruments (e.g., COSMIN [35]) or diagnostic test accuracy studies (e.g., QAREL [36]). As GRRAS was originally developed as a reporting guideline, its items will be adapted for the purpose of methodological quality appraisal, focusing on aspects with direct implications for the interpretation of reliability and agreement estimates, including participant selection and spectrum, assessor selection and expertise, standardisation of the assessment procedure, independence of ratings, timing of assessments, handling of missing or indeterminate data, appropriateness of the statistical method used to quantify reliability or agreement and completeness of reporting. The specific criteria used to rate each domain as low concern, high concern, or unclear concern are detailed in Appendix 3. The checklist is intended to identify methodological and reporting concerns relevant to the interpretation of inter‐rater reliability, agreement or variability estimates, rather than to generate a single overall judgement. If any single domain is rated as high concern, the study will be classified as high concern.

Disagreements between reviewers will be resolved through discussion and, if necessary, consultation with a third reviewer. The results of the methodological quality assessment will be presented descriptively and considered in the interpretation of the review findings.

### 2.9 Classification of Composite Airway Assessment Tools

Composite airway assessment tools identified in the included studies will be described according to their component variables, scoring structure and intended clinical use. A preliminary overview of selected tools and their components is provided in Appendix 2. Additional tools identified during the review process will be added to the extraction table and described in the final review where relevant.

### 2.10 Reliability Estimates and Reporting Modalities

Studies may report inter‐rater estimates as variability, agreement or reliability. These terms are often used interchangeably but may be quantified using different statistical approaches depending on the type of assessment, number of assessors and scale of measurement, including Cohen's kappa [21], weighted kappa [22], Fleiss’ kappa [23], percentage agreement, intraclass correlation coefficients (ICC) [24], Bland–Altman analysis [25], Gwet's AC1 or AC2 [26] or Spearman's or Pearson's correlation coefficient [27, 28]. For each included study, the reported measure, estimate, confidence interval where available and relevant assessor characteristics will be extracted; where more than one measure is reported for the same tool or component, all will be extracted and presented descriptively.

For categorical or ordinal outcomes, chance‐corrected measures (e.g., kappa [21], weighted kappa [22], Gwet's AC1 [26]) will be prioritised over uncorrected percentage agreement; for continuous or approximately continuous scores, ICC [24] will be prioritised. Where a study reports more than one chance‐corrected measure, the one most consistently reported across included studies for a given tool or component will be prioritised for quantitative synthesis, to maximise comparability; this decision and its rationale will be documented transparently in the final review. Percentage agreement will be presented descriptively and not pooled unless no chance‐corrected estimate is available, and studies are otherwise highly comparable.

Correlation coefficients (Spearman's or Pearson's [27, 28]) will be extracted when reported by included studies but will not be treated as measures of reliability or agreement, as correlation and agreement are distinct statistical concepts that can diverge substantially in practice [31, 32, 33, 34, 35, 36, 37]; notably, correlation is unaffected by systematic bias between assessors, meaning two assessors could show perfect correlation despite consistently disagreeing on the absolute score [31]. Correlation estimates will be synthesised separately from, and will not be pooled together with, agreement or reliability estimates. Where intraclass correlation coefficients are reported, their potential sensitivity to sample heterogeneity will be considered when interpreting reliability estimates across studies with differing participant spectra [32, 37].

Estimates derived from different statistical approaches are not directly comparable and will be grouped by statistical measure, assessment type and population type. Where a single study contributes multiple estimates for the same tool or component (e.g., from different assessor pairs, groups, or populations), these will not be treated as statistically independent; only one estimate per study per tool/component and population subgroup will enter a given pooled analysis, with remaining estimates reported narratively. Quantitative synthesis may be considered where two or more studies report a comparable estimate for the same tool or component within a comparable population and are judged sufficiently homogeneous; otherwise, findings will be synthesised narratively.

### 2.11 Data Synthesis

Findings will primarily be synthesised narratively using descriptive tables and summaries, reflecting the anticipated methodological and clinical heterogeneity of the included literature. Included studies will be summarised by key characteristics, including study design, patient population, clinical setting, assessor characteristics and type of assessment evaluated. Studies will be grouped by type of assessment (composite tools vs. individual components) and stratified by population type (clinical perioperative populations vs. healthy volunteers or research/training settings). Reported reliability, agreement or variability estimates will be presented alongside relevant characteristics, including assessor profession or experience, timing and conditions of assessment, assessment method and independence or blinding of ratings.

Quantitative synthesis may be considered where two or more studies report a comparable statistical measure for the same tool or component within a comparable population, and only where studies are judged sufficiently homogeneous with respect to population, assessor characteristics, assessment procedures and statistical methodology. Where performed, a sensitivity analysis may compare pooled estimates with and without studies judged at high concern (Appendix 3). Where quantitative synthesis is not appropriate or possible, which is expected for most assessment types given the anticipated heterogeneity, findings will instead be synthesised narratively, with consideration of methodological quality, clinical heterogeneity and consistency across studies.

Formal assessment of publication bias will only be performed if sufficient comparable studies are included in a quantitative synthesis; otherwise, its potential influence will be considered narratively. The methodological quality assessment (Appendix 3) will likewise inform interpretation of the review findings. The overall certainty in the evidence for each tool or component will be considered narratively, based on the number and methodological quality of contributing studies (Appendix 3), the consistency of reliability estimates across studies, and the precision of pooled estimates were reported.

## 3 Discussion

### 3.1 Rationale and Relevance

Airway assessment is routinely used in pre‐anaesthetic care to support perioperative planning, airway management strategies and communication of risk [2, 15]. Because these assessments may influence clinical decisions across a wide range of surgical settings, their consistency between assessors is clinically relevant.

Such assessments rely on clinical judgement and interpretation, whether through direct physical examination, such as Mallampati classification [3] or thyromental distance [4], or through composite scoring systems that combine multiple such components [6, 7, 8, 9, 10]. Variation in how they are performed or interpreted may affect risk stratification, communication between clinicians, and decisions regarding airway management, monitoring, and allocation of experienced personnel [11, 12]. Inter‐rater reliability is therefore an important consideration when evaluating the clinical usefulness of these routinely used tools.

This systematic review will synthesise published studies reporting inter‐rater reliability, agreement or variability in individual components and composite tools. It may clarify how agreement has been quantified, which methods and assessor groups have been studied and how reported agreement varies across clinical settings, populations and methods, contributing to a more informed understanding of their consistency and helping identify areas for further methodological research, clearer guidance or standardisation.

More broadly, robust estimates of human inter‐rater reliability may serve a purpose beyond characterising current practice. As automated and AI‐supported approaches continue to emerge [38], such estimates may provide a reference point for evaluating whether these technologies achieve consistency comparable to routine clinical assessment under real‐world conditions.

### 3.2 Strengths and Limitations

This protocol's methodology is systematic and transparent, developed in accordance with PRISMA‐P [17], PRISMA 2020 [18] and the Cochrane Handbook for Systematic Reviews [19], with predefined eligibility criteria and independent duplicate screening, extraction, and quality assessment intended to improve reproducibility and reduce reviewer‐dependent bias.

Several limitations should nonetheless be considered. Clinical and methodological heterogeneity across included studies is expected, given likely differences in population, setting, assessor profession and experience and statistical methods; this may limit comparability and reduce the feasibility of quantitative synthesis. Restricting inclusion to peer‐reviewed full‐text publications, chosen to ensure sufficient detail for eligibility assessment and data extraction, may also exclude relevant data from grey literature, contributing to a degree of publication‐format bias.

## 4 Conclusion

This systematic review will identify, assess and synthesise the available literature on inter‐rater reliability, agreement or variability in individual airway assessment components and composite tools in the context of pre‐anaesthetic assessment. The findings may contribute to a better understanding of how consistently these commonly used methods are applied and help identify areas where further methodological research or standardisation may be relevant.

## Author Contributions

All authors (Constance Thyra Giertz Christiansen, Mathilde Nellemann Jensen, Moritz Kilian German Denneborg, Sofie Amalie Bosholdt, Janus Christian Jakobsen, Rasmus Tingkær Hesselfeldt, Morten Hylander Møller, Andreas Møgelmose, Markus Harboe Olsen, Michael Seltz Kristensen and Katrine Bredahl Buggeskov) contributed to the conception and design of the protocol. Constance Thyra Giertz Christiansen drafted the manuscript. All authors critically revised the manuscript for important intellectual content, approved the final version to be published and agree to be accountable for all aspects of the work in ensuring that questions related to the accuracy or integrity of any part of the work are appropriately investigated and resolved.

## Funding

No specific external funding has been received for this systematic review protocol. All authors will receive salary funding from their respective departments.

## Conflicts of Interest

The authors declare no conflicts of interest.

## Data Availability

No new data were generated or analysed for this protocol. The eligibility criteria, data extraction items and full search strategies to be used in the systematic review are provided in the protocol and its appendices. Following completion of the systematic review, the extracted data, the completed data extraction forms and the methodological quality assessments will be made available from the corresponding author upon reasonable request.

## Appendix 1 Search Strategy

### Embase

**Table:**

Step	Search terms	Results
	**Concept 1**	
1	mallampati.ab,kf,ti.	2503
2	thyromental.ab,kf,ti.	884
3	(mouth adj5 (open* or close*)).ab,kf,ti.	13,177
4	interincisal distance.ab,kf,ti.	355
5	((head or jaw or neck or cervical) adj5 (move* or moving or mobil*)).ab,kf,ti.	25,515
6	prognat*.ab,kf,ti.	2848
7	jaw thrust.ab,kf,ti.	525
8	upper lip bite test*.ab,kf,ti.	239
9	ULBT.ab,kf,ti.	114
10	((Airway* or intubat*) adj3 (difficult or assessment or obstruct* or evaluat*)).ab,kf,ti.	64,693
11	BMI.ab,kf,ti.	536,687
12	body mass index.ab,kf,ti.	439,635
13	exp overweight/	846,208
14	obesity.ab,kf,ti.	621,415
15	obese.ab,kf,ti.	281,436
16	SARI.ab,kf,ti.	2860
17	simplified airway risk index.ab,kf,ti.	16
18	egri.ab,kf,ti.	50
19	(El‐Ganzouri adj3 (index or score or risk)).ab,kf,ti.	44
20	(Wilson adj3 score).ab,kf,ti.	363
21	receding mandible.ab,kf,ti.	41
22	buck teeth.ab,kf,ti.	10
23	mandibular subluxation.ab,kf,ti.	41
24	lemon scor*.ab,kf,ti.	20
25	macocha.ab,kf,ti.	32
26	IIG.ab,kf,ti.	304
27	inter‐incisor gap.ab,kf,ti.	75
28	hyomental distance.ab,kf,ti.	135
29	HMD.ab,kf,ti.	2290
30	(sternomental adj5 distance).ab,kf,ti.	266
31	SMD.ab,kf,ti.	32,030
32	neck circumference.ab,kf,ti.	4536
33	mandibular length.ab,kf,ti.	839
34	temporomandibular joint.ab,kf,ti.	23,233
35	TMJ.ab,kf,ti.	14,610
36	retrognath*.ab,kf,ti.	2453
37	retrogenia.ab,kf,ti.	39
38	obstructive sleep apnea.ab,kf,ti.	66,488
39	OSA.ab,kf,ti.	49,689
40	arne scor*.ab,kf,ti.	12
41	naguib.ab,kf,ti.	59
42	exp airway obstruction/	53,191
43	1 or 2 or 3 … or 42	1,688,023

Step	Search terms	Results
	**Concept 2**	
44	preanaesthe*.ab,kf,ti.	570
45	preanesthe*.ab,kf,ti.	2107
46	exp anesthesia/	489,270
47	Preoperative care/	53,064
48	anaesthe*.ab,kf,ti.	191,910
49	anesthe*.ab,kf,ti.	487,045
50	preopera*.ab,kf,ti.	622,045
51	intubat*.ab,kf,ti.	143,230
52	exp preoperative evaluation/	197,789
53	exp endotracheal intubation/	79,227
54	44 or 45 or 46 … or 53	1,582,691

Step	Search terms	Results
	**Concept 3**	
55	interrater reliability/	45,965
56	observer variation/	24,078
57	interrater variab*.ab,kf,ti.	498
58	inter‐rater variab*.ab,kf,ti.	1126
59	agreement.ab,kf,ti.	471,145
60	interobserver variability.ab,kf,ti.	8546
61	inter‐observer variability.ab,kf,ti.	5658
62	observer variation.ab,kf,ti.	2699
63	reproducibility.ab,kf,ti.	155,181
64	kappa.ab,kf,ti.	171,207
65	intraclass correlation coefficients.ab,kf,ti.	16,536
66	intraclass correlation.ab,kf,ti.	51,713
67	variability.ab,kf,ti.	575,221
68	reliability.ab,kf,ti.	356,517
69	validity.ab,kf,ti.	325,956
70	bland–altman.ab,kf,ti.	38,098
71	limits of agreement.ab,kf,ti.	18,130
72	((spearman or pearson) adj correlation).ab,kf,ti.	92,749
73	gwet.ab,kf,ti.	215
74	AC1.ab,kf,ti.	1754
75	first‐order agreement coefficient.ab,kf,ti.	58
76	concordance.ab,kf,ti.	122,222
77	55 or 56 or 57 … or 76	1,921,855

	Concept 1 and Concept 2 and Concept 3	
78	43 and 54 and 77	4652

### Medline

**Table:**

Step	Search terms	Results
	Concept 1	
1	mallampati.ab,kf,ti.	1148
2	thyromental.ab,kf,ti.	430
3	(mouth adj5 (open* or close*)).ab,kf,ti.	10,337
4	interincisal distance.ab,kf,ti.	289
5	((head or jaw or neck or cervical) adj5 (move* or moving or mobil*)).ab,kf,ti.	18,786
6	prognat*.ab,kf,ti.	2949
7	jaw thrust.ab,kf,ti.	266
8	‘upper lip bite test*’.ab,kf,ti.	119
9	ULBT.ab,kf,ti.	53
10	((Airway* or intubat*) adj3 (difficult or assessment or obstruct* or evaluat*)).ab,kf,ti.	41,784
11	BMI.ab,kf,ti.	254,892
12	body mass index.ab,kf,ti.	296,206
13	exp overweight/	310,627
14	obese.ab,kf,ti.	171,644
15	SARI.ab,kf,ti.	1526
16	simplified airway risk index.ab,kf,ti.	7
17	egri.ab,kf,ti.	60
18	(El‐Ganzouri adj3 (index or score or risk)).ab,kf,ti.	20
19	(Wilson adj3 score).ab,kf,ti.	203
20	receding mandible.ab,kf,ti.	20
21	buck teeth.ab,kf,ti.	2
22	mandibular subluxation.ab,kf,ti.	34
23	lemon scor*.ab,kf,ti.	14
24	macocha.ab,kf,ti.	14
25	IIG.ab,kf,ti.	203
26	inter‐incisor gap.ab,kf,ti.	38
27	hyomental distance.ab,kf,ti.	80
28	HMD.ab,kf,ti.	1752
29	(sternomental adj5 distance).ab,kf,ti.	135
30	SMD.ab,kf,ti.	27,384
31	neck circumference.ab,kf,ti.	2293
32	mandibular length.ab,kf,ti.	804
33	temporomandibular joint*.ab,kf,ti.	22,484
34	TMJ.ab,kf,ti.	12,437
35	retrognath*.ab,kf,ti.	1954
36	retrogenia.ab,kf,ti.	29
37	obstructive sleep apnea.ab,kf,ti.	38,715
38	OSA.ab,kf,ti.	26,336
39	arne scor*.ab,kf,ti.	8
40	naguib.ab,kf,ti.	19
41	1 or 2 or 3 … or 40	815,254

Step	Search terms	Results
	Concept 2	
42	‘preanaesthe*’.ab,kf,ti.	354
43	‘preanesthe*’.ab,kf,ti.	2102
44	exp anesthesia/	218,183
45	Preoperative care/	69,292
46	‘anaesthe*’.ab,kf,ti.	125,165
47	‘anesthe*’.ab,kf,ti.	365,399
48	‘preopera*’.ab,kf,ti.	445,245
49	intubat*.ab,kf,ti.	79,762
50	exp Intubation, Intratracheal/	45,504
51	42 or 43 or 44 … or 50	1,051,952

Step	Search terms	Results
	Concept 3	
52	‘Reproducibility of results’/	513,455
53	observer variation/	47,377
54	interrater variab*.ab,kf,ti.	310
55	‘inter‐rater variab*’.ab,kf,ti.	609
56	agreement.ab,kf,ti.	380,067
57	interobserver variability.ab,kf,ti.	5782
58	inter‐observer variability.ab,kf,ti.	2849
59	observer variation.ab,kf,ti.	1708
60	reproducibility.ab,kf,ti.	121,409
61	kappa.ab,kf,ti.	138,914
62	Intraclass correlation coefficients.ab,kf,ti.	14,152
63	intraclass correlation.ab,kf,ti.	42,808
64	‘variability’.ab,kf,ti.	430,434
65	‘reliability’.ab,kf,ti.	287,582
66	validity.ab,kf,ti.	263,476
67	‘bland–altman’.ab,kf,ti.	26,114
68	‘limits of agreement’.ab,kf,ti.	13,469
69	((spearman or pearson) adj correlation).ab,kf,ti.	56,709
70	gwet.ab,kf,ti.	200
71	‘AC1’.ab,kf,ti.	1413
72	‘first‐order agreement coefficient’.ab,kf,ti.	53
73	concordance.ab,kf,ti.	77,558
74	52 or 53 or 54 … or 73	1,764,802

	Concept 1 and Concept 2 and Concept 3	
75	41 and 51 and 74	2380

### Cochrane

**Table:**

Step	Search terms	Results
	Concept 1	
1	(mallampati):ti,ab,kw	958
2	(thyromental):ti,ab,kw	203
3	(mouth NEXT (open* or close*)):ti,ab,kw	2731
4	(interincisal distance):ti,ab,kw	220
5	(((head or jaw or neck or cervical) NEAR/5 (move* or moving or mobil*))):ti,ab,kw	3918
6	(prognat*):ti,ab,kw	174
7	(jaw thrust):ti,ab,kw	306
8	(‘upper lip bite test’):ti,ab,kw	30
9	(ULBT):ti,ab,kw	9
10	(((Airway* or intubat*) NEAR/3 (difficult or assessment or obstruct* or evaluat*))):ti,ab,kw	7526
11	(BMI):ti,ab,kw	75,143
12	(‘body‐mass index’):ti,ab,kw	50,459
13	MeSH descriptor: [Overweight] explode all trees	27,697
14	(obese):ti,ab,kw	30,121
15	(SARI):ti,ab,kw	297
16	(simplified airway risk index):ti,ab,kw	16
17	(EGRI):ti,ab,kw	13
18	(El‐Ganzouri NEAR/3 (index or score or risk)):ti,ab,kw	26
19	(wilson NEAR/3 score):ti,ab,kw	36
20	(receding mandible):ti,ab,kw	4
21	(buck teeth):ti,ab,kw	4
22	(mandibular subluxation):ti,ab,kw	6
23	(lemon scor*):ti,ab,kw	196
24	(macocha):ti,ab,kw	2
25	(IIG):ti,ab,kw	41
26	(inter‐incisor gap):ti,ab,kw	13
27	(hyomental distance):ti,ab,kw	20
28	(HMD):ti,ab,kw	299
29	(sternomental NEAR/3 distance):ti,ab,kw	51
30	(SMD):ti,ab,kw	1689
31	(neck circumference):ti,ab,kw	634
32	(mandibular length):ti,ab,kw	697
33	(TMJ):ti,ab,kw	1178
34	(retrognath*):ti,ab,kw	199
35	(retrogenia):ti,ab,kw	1
36	(temporomandibular joint*):ti,ab,kw	2754
37	(obstructive sleep apnea):ti,ab,kw	7866
38	(OSA):ti,ab,kw	5051
39	(arne scor*):ti,ab,kw	14
40	(naguib):ti,ab,kw	7
41	#1 or #2 or #3 or #4 or #5 or #6 or #7 or #8 or #9 or #10 or #11 or #12 or #13 or #14 or #15 or #16 or #17 or #18 or #19 or #20 or #21 or #22 or #23 or #24 or #25 or #26 or #27 or #28 or #29 or #30 or #31 or #32 or #33 or #34 or #35 or #36 or #37 or #38 or #39 or #40	151,764

Step	Search terms	Results
	Concept 2	
42	(preanesthe*):ti,ab,kw	2735
43	(preanaesthe*):ti,ab,kw	403
44	(preopera*):ti,ab,kw	65,743
45	(intubat*):ti,ab,kw	30,998
46	MeSH descriptor: [Intubation, Intratracheal] explode all trees	5844
47	#42 or #43 or #44 or #45 or #46	96,756

Step	Search terms	Results
	Concept 3	
48	MeSH descriptor: [Reproducibility of Results] explode all trees	16,978
49	MeSH descriptor: [Observer Variation] explode all trees	2705
50	(interrater variab*):ti,ab,kw	492
51	(inter‐rater variab*):ti,ab,kw	260
52	(agreement):ti,ab,kw	15,342
53	(inter‐observer variability):ti,ab,kw	255
54	(interobserver variability):ti,ab,kw	624
55	(observer variation):ti,ab,kw	3075
56	(reproducibility):ti,ab,kw	19,602
57	(kappa):ti,ab,kw	5595
58	(Intraclass correlation coefficient*):ti,ab,kw	3239
59	(Intraclass correlation):ti,ab,kw	3700
60	(vaiability):ti,ab,kw	2
61	(reliability):ti,ab,kw	16,390
62	(validity):ti,ab,kw	16,248
63	(bland–altman):ti,ab,kw	1558
64	(limits of agreement):ti,ab,kw	1164
65	((spearman or pearson) NEXT correlation):ti,ab,kw	6141
66	(gwet):ti,ab,kw	47
67	(AC1):ti,ab,kw	108
68	(first‐order agreement coefficient):ti,ab,kw	7
69	(concordance):ti,ab,kw	4490
70	#48 or #49 or #50 or #51 or #52 or #53 or #54 or #55 or #56 or #57 or #58 or #59 or #60 or #61 or #62 or #63 or #64 or #65 or #66 or #67 or #68 or #69	65,377

	Concept 1 and Concept 2 and Concept 3	
71	#41 and #47 and #70 in Trials	245

### CINAHL

**Table:**

Step	Search terms	Results
	Concept 1	
1	MH ‘Obesity+’	
2	XB (mallampati)	
3	XB (thyromental)	
4	XB ((mouth N5 (open* or close*)))	
5	XB (interincisal distance)	
6	XB (((head or jaw or neck or cervical) N5 (move* or moving or mobil*)))	
7	XB (prognat*)	
8	XB (jaw thrust)	
9	XB (‘upper lip bite test*’)	
10	XB (ULBT)	
11	XB (((Airway* or intubat*) N3 (difficult or assessment or obstruct* or evaluat*)))	
12	XB (BMI)	
13	XB (body mass index)	
14	XB (obese)	
15	XB (SARI)	
16	XB (simplified airway risk index)	
17	XB (egri)	
18	XB ((El‐Ganzouri N3 (index or score or risk)))	
19	XB ((Wilson N3 score))	
20	XB (receding mandible)	
21	XB (buck teeth)	
22	XB (mandibular subluxation)	
23	XB (lemon scor*)	
24	XB (macocha)	
25	XB (IIG)	
26	XB (inter‐incisor gap)	
27	XB (hyomental distance)	
28	XB (HMD)	
29	XB (sternomental N5 distance)	
30	XB (SMD)	
31	XB (neck circumference)	
32	XB (mandibular length)	
33	XB (temporomandibular joint*)	
34	XB (TMJ)	
35	XB (retrognath*)	
36	XB (retrogenia)	
37	XB (obstructive sleep apnea)	
38	XB (OSA)	
39	XB (arne scor*)	
40	XB (naguib)	
41	CONCEPT 1 – combined with OR	261,268

Step	Search terms	Results
	Concept 2	
42	XB (‘preanaesthe*’)	
43	XB (preanesthe)	
44	MH ‘Anesthesia+’	
45	MH ‘Preoperative Care+’	
46	XB (anaesthe*)	
47	XB (anesthe*)	
48	XB (preopera*)	
49	XB (intubat*)	
50	MM ‘Intubation, Intratracheal+’	
51	Concept 2—combined with OR	224,733

Step	Search terms	Results
	Concept 3	
52	MM ‘Reproducibility of Results’	16,978
53	MH ‘Observer Bias+’	2705
54	MH ‘Interrater Reliability’	492
55	MH ‘Consensus’	260
56	MH ‘kappa Statistic’	15,342
57	XB (interrater variab*)	255
58	XB (‘inter‐rater variab*’)	624
59	XB (agreement or reliability or inter‐rater reliability or accuracy or icc or kappa or scoring or grade or mark)	3075
60	XB (interobserver variability)	19,602
61	XB (inter‐observer variability)	5595
62	XB (observer variation)	3239
63	XB (reproducibility)	3700
64	XB (Intraclass correlation coefficients)	2
65	XB (intraclass correlation)	16,390
66	XB (variability)	16,248
67	XB (reliability)	1558
68	XB (validity)	1164
69	XB (bland–altman)	6141
70	XB (gwet)	47
71	MH ‘Spearman's Rank Correlation Coefficient’	108
72	XB (AC1)	7
73	XB (concordance)	4490
74	Concept 3 – combined with OR	587,853

	Concept 1 and Concept 2 and Concept 3	
75	Concept 1 and Concept 2 and Concept 3	2005

## Appendix 2 Composite Airway Assessment Tools and Their Scoring Systems

### SARI [20]

**Table:**

Simplified airway risk index (SARI)	Total of 0–12 points
Mallampati classification	I → 0 points
	II → 0 points
	III → 1 point
	IV → 2 points
Neck movement	> 90° flexion and extension → 0 points
	80°–90° flexion or extension → 1 point
	< 80° extension or extension → 2 points
Mouth opening	≥ 4 cm → 0 points
	< 4 cm → 1 point
Thyromental distance	> 6.5 cm → 0 points
	6.0–6.5 → 1 point
	< 6.5 cm → 2 points
Ability to prognath	Yes → 0 points
	No → 1 point
Weight	< 90 kg → 0 points
	90–110 kg → 1 point
	> 110 kg → 2 points
History of difficult intubation	Absent → 0 points
	Questionable → 1 point
	Definite → 2 points

*Note:* Mallampati's original description [3] comprised three classes; a fourth class was later added by Samsoon and Young [39]. SARI is shown here as operationalised by Nørskov et al. [20], which differs slightly in Mallampati weighting from El‐Ganzouri's original composite model [6], from which EGRI below is drawn directly. Where included studies report a different classification or weighting, this will be recorded as reported.

### EGRI [6]

**Table:**

El‐Ganzouri risk index (EGRI)	Total of 0–12 points
Mallampati classification	I → 0 points
	II → 1 point
	III → 2 points
Neck movement	> 90° → 0 points
	80°–90° → 1 point
	< 80° → 2 points
Mouth opening	≥ 4 cm → 0 points
	< 4 cm → 1 point
Thyromental distance	> 6,5 cm → 0 points
	6.0–6.5 → 1 point
	<6,0 cm → 2 points
Teeth prognathism	Yes → 0 points
	No → 1 point
Weight	< 90 kg → 0 points
	90–110 kg → 1 point
	> 110 kg → 2 points
Previous difficult intubation	Absent → 0 points
	Questionable → 1 point
	Definite → 2 points

### Wilson [4]

**Table:**

Wilson score	Total of 0–10 points
Jaw movement	Inter‐incisor gap > 5 cm or mandibular subluxation > 0 → 0 points
	Inter‐incisor gap < 5 cm or mandibular subluxation = 0 → 1 point
	Inter‐incisor gap < 5 cm or mandibular subluxation < 0 → 2 points
Head and neck movement	> 90° → 0 points
	90° → 1 point
	< 90° → 2 points
Receding mandible	Normal → 0 points
	Moderate → 1 point
	Severe → 2 points
Buck teeth	Normal → 0 points
	Moderate → 1 point
	Severe → 2 points
Weight	< 90 kg → 0 points
	90–110 kg → 1 point
	> 110 kg → 2 points

### LEMON [7]

**Table:**

LEMON score	Total of 0–5 points
Look externally	Normal facial/airway anatomy → 0 points
	Abnormal (Visible signs of difficult airway, e.g., facial trauma, large incisors, beard, large tongue) → 1 point
Evaluate 3‐3‐2	Mouth opening ≥ 3 fingerbreadths, hyomental distance ≥ 3 fingerbreadths, thyroid‐to‐floor‐of‐mouth distance ≥ 2 fingerbreadths → 0 points
	Any distance below threshold → 1 point
Mallampati	I–II → 0 points
	III–IV → 1 point
Obstruction	No signs of upper airway obstruction → 0 points
	Signs of obstruction (stridor, epiglottitis, etc.) → 1 point
Neck mobility	Full range of neck movement → 0 points
	Limited neck movement → 1 point

### MACOCHA [8]

**Table:**

MACOCHA score	Total of 0–12 points
Mallampati	I → 0 points
	II → 0 points
	III → 5 points
	IV → 5 points
Obstructive sleep apnoea	Absent → 0 points
	Present → 2 points
Cervical spine mobility	Normal → 0 points
	Reduced → 1 point
Mouth opening	≥ 3 cm → 0 points
	< 3 cm → 1 point
Coma	Absent → 0 points
	Present → 1 point
Hypoxia	SpO_2_ ≥ 80% → 0 points
	SpO_2_ < 80% → 1 point
Operator	Anaesthesiologist → 0 points
	Non‐anaesthesiologist → 1 point

### ARNÉ [9]

**Table:**

ARNÉ score	> 11 points indicate predicted difficult intubation
Previous history of difficult intubation	No → 0 points
	Yes → 10 points
Pathologies associated with difficult intubation	No → 0 points
	Yes → 5 points
Clinical symptoms of airway pathology	No → 0 points
	Yes → 3 points
Inter‐incisor gap (IG) and mandibular luxation (ML)	IG > 5 cm or ML > 0 → 0 points
	IG 3.5–5 cm and ML = 0 → 3 points
	IG < 3.5 cm and ML < 0 → 13 points
Thyromental distance	≥6.5 cm → 0 points
	<6.5 cm → 4 points
Maximum range of head and neck movement	Above 100° → 0 points
	About 90° (90° ± 10°) → 2 points
	Below 80° → 5 points
Mallampati's modified test	Class I → 0 points
	Class II → 2 points
	Class III–IV → 6 points

### Naguib [10]

**Table:**

Naguib score	Logistic regression equation
	Prediction (l) = 0.2262−0.4621 × thyromentaldistance +2.5516 × Mallampatiscore −1.1461 × interincisorgap + 0.0433 × height
	Predicted value < 0 → easy intubation anticipated
	Predicted value > 0 → difficult intubation anticipated
Thyromental distance	Continuous variable in cm
Mallampati classification	I–II → 0 points
	III–IV → 1 point
Inter‐incisor gap	Continuous variable in cm
Height	Continuous variable in cm

*Note:* Unlike the tools above, the Naguib model [10] is a logistic regression equation rather than an additive point system. It has not been independently prospectively validated. An earlier, distinct four‐variable ‘Naguib model’ was described in a separate 1999 publication by the same first author [40]; the specific variables and equation used will therefore be recorded for each included study.

## Appendix 3 Operationalised Domains for Methodological Quality Assessment

Each included study will be assessed across the following domains. Ratings will be assigned as low concern, high concern, or unclear concern for each domain. The checklist is intended to identify methodological and reporting concerns relevant to the interpretation of inter‐rater reliability, agreement or variability estimates, rather than to generate a single overall judgement.

**Table:**

Domain	What is assessed	Low concern	High concern	Unclear
Participant selection/spectrum	Is the participant population clearly described and appropriate for the stated study setting and intended interpretation?	Participant characteristics and setting are clearly reported and the spectrum is appropriate for the stated purpose of the study.	Highly selected or restricted population that is not appropriate for the stated purpose or selection likely to distort agreement estimates.	Participant spectrum or selection process not sufficiently reported.
Assessor selection and expertise	Are the number, profession and experience of assessors clearly described and appropriate for the study purpose?	Number of assessors and relevant assessor characteristics are clearly reported and appropriate for the stated study purpose.	Assessor selection is likely to distort agreement estimates, such as assessors being unusually trained or selected in a way inconsistent with the intended use, without acknowledgement or analysis.	Assessor characteristics not sufficiently reported.
Standardisation of assessment procedure	Were assessment definitions, scoring categories and procedures sufficiently standardised across assessors?	Assessment procedure, definitions, categories and instructions are clearly described and applied consistently.	Assessors used different procedures, definitions or scoring criteria, or no attempt was made to standardise the assessment where standardisation was required.	Standardisation or assessor instructions not reported.
Independence of ratings	Were ratings made independently, without discussion or knowledge of other assessors' ratings?	Independent ratings are explicitly reported and plausible.	Assessors were aware of each other's ratings, discussed ratings before recording, or independence was not maintained.	Independence of ratings not reported.
Timing between ratings	Was the timing between ratings appropriate for a stable assessment condition?	Ratings were performed during the same encounter or within a short interval, with no plausible change in participant status.	The time interval was long enough for participant status or assessment conditions to plausibly change, or timing/assessment sequence introduced a risk of influence between assessors.	Timing between ratings not reported.
Handling of missing/indeterminate ratings	Were missing, excluded or indeterminate ratings reported and handled transparently?	Missing or indeterminate ratings are reported, explained and handled appropriately.	Substantial missing or excluded ratings are unexplained, selectively omitted or handled in a way likely to bias estimates.	Missing data handling not reported.
Appropriateness of statistical method	Was the statistical method appropriate for the data type, number of raters, scale of measurement, and study design?	Statistical method is appropriate and sufficiently specified, including weighting or ICC type where relevant.	Clear methodological misapplication, such as treating correlation as agreement, using a two‐rater method for multiple raters without justification, or using an inappropriate method for the scale of measurement.	Statistical method insufficiently described to judge.
Completeness of reporting/interpretability	Are the reliability/agreement estimates reported with sufficient information for interpretation?	Estimate, measure of precision where reported, number of ratings/participants, and relevant context are reported.	Reporting is insufficient for meaningful interpretation, such as a point estimate only with no denominator, no description of raters or unclear assessment categories.	Partial reporting, with some but not all necessary contexts.

## Appendix 4 PRISMA‐P Checklist [17]

**Table:**

Section and topic	Item no	Checklist item	Reported in protocol
Administrative information			
Title: Identification	1a	Identify the report as a protocol of a systematic review	X
Title: Update	1b	If the protocol is for an update of a previous systematic review, identify as such	X
Registration	2	If registered, provide the name of the registry (such as PROSPERO) and registration number	X
Authors: Contact	3a	Provide name, institutional affiliation, e‐mail address of all protocol authors; provide physical mailing address of corresponding author	X
Authors: Contributions	3b	Describe contributions of protocol authors and identify the guarantor of the review	X
Amendments	4	If the protocol represents an amendment of a previously completed or published protocol, identify as such and list changes; otherwise, state plan for documenting important protocol amendments	X
Support: Sources	5a	Indicate sources of financial or other support for the review	X
Support: Sponsor	5b	Provide name for the review funder and/or sponsor	X
Support: Role of sponsor or funder	5c	Describe roles of funder(s), sponsor(s), and/or institution(s), if any, in developing the protocol	X
Introduction			
Rationale	6	Describe the rationale for the review in the context of what is already known	X
Objectives	7	Provide an explicit statement of the question(s) the review will address with reference to participants, interventions, comparators, and outcomes (PICO)	X
Methods			
Eligibility criteria	8	Specify the study characteristics (such as PICO, study design, setting, time frame) and report characteristics (such as years considered, language, publication status) to be used as criteria for eligibility for the review	X
Information sources	9	Describe all intended information sources (such as electronic databases, contact with study authors, trial registers or other grey literature sources) with planned dates of coverage	X
Search strategy	10	Present draft of search strategy to be used for at least one electronic database, including planned limits, such that it could be repeated	X
Study records: Data management	11a	Describe the mechanism(s) that will be used to manage records and data throughout the review	X
Study records: Selection process	11b	State the process that will be used for selecting studies (such as two independent reviewers) through each phase of the review (i.e., screening, eligibility and inclusion in meta‐analysis)	X
Study records: Data collection process	11c	Describe planned method of extracting data from reports (such as piloting forms, done independently, in duplicate), any processes for obtaining and confirming data from investigators	X
Data items	12	List and define all variables for which data will be sought (such as PICO items, funding sources), any pre‐planned data assumptions and simplifications	X
Outcomes and prioritization	13	List and define all outcomes for which data will be sought, including prioritization of main and additional outcomes, with rationale	X
Risk of bias in individual studies	14	Describe anticipated methods for assessing risk of bias of individual studies, including whether this will be done at the outcome or study level, or both; state how this information will be used in data synthesis	X
Data synthesis	15a	Describe criteria under which study data will be quantitatively synthesised	X
	15b	If data are appropriate for quantitative synthesis, describe planned summary measures, methods of handling data and methods of combining data from studies, including any planned exploration of consistency (such as I^2^, Kendall's τ)	X
	15c	Describe any proposed additional analyses (such as sensitivity or subgroup analyses, meta‐regression)	X
	15d	If quantitative synthesis is not appropriate, describe the type of summary planned	X
Meta‐bias(es)	16	Specify any planned assessment of meta‐bias(es) (such as publication bias across studies, selective reporting within studies)	X
Confidence in cumulative evidence	17	Describe how the strength of the body of evidence will be assessed (such as GRADE)	X

## Appendix 5 PRISMA Checklist [18]

As this document is a protocol, the PRISMA checklist cannot yet be completed in full; it will be completed and provided as supplementary material alongside the final systematic review report.

**Table:**

Section and topic	Item #	Checklist item
Title	1	Identify the report as a systematic review.
Abstract	2	See the PRISMA 2020 for Abstracts checklist.
Rationale	3	Describe the rationale for the review in the context of existing knowledge.
Objectives	4	Provide an explicit statement of the objective(s) or question(s) the review addresses.
Eligibility criteria	5	Specify the inclusion and exclusion criteria for the review and how studies were grouped for the syntheses.
Information sources	6	Specify all databases, registers, websites, organisations, reference lists and other sources searched or consulted to identify studies. Specify the date when each source was last searched or consulted.
Search strategy	7	Present the full search strategies for all databases, registers and websites, including any filters and limits used.
Selection process	8	Specify the methods used to decide whether a study met the inclusion criteria of the review, including how many reviewers screened each record and each report retrieved, whether they worked independently and if applicable, details of automation tools used in the process.
Data collection process	9	Specify the methods used to collect data from reports, including how many reviewers collected data from each report, whether they worked independently, any processes for obtaining or confirming data from study investigators, and if applicable, details of automation tools used in the process.
Data items	10a	List and define all outcomes for which data were sought. Specify whether all results that were compatible with each outcome domain in each study were sought and if not, the methods used to decide which results to collect.
	10b	List and define all other variables for which data were sought (e.g., participant and intervention characteristics, funding sources). Describe any assumptions made about any missing or unclear information.
Study risk of bias assessment	11	Specify the methods used to assess risk of bias in the included studies, including details of the tool(s) used, how many reviewers assessed each study and whether they worked independently and if applicable, details of automation tools used in the process.
Effect measures	12	Specify for each outcome the effect measure(s) (e.g., risk ratio, mean difference) used in the synthesis or presentation of results.
Synthesis methods	13a	Describe the processes used to decide which studies were eligible for each synthesis.
	13b	Describe any methods required to prepare the data for presentation or synthesis, such as handling of missing summary statistics, or data conversions.
	13c	Describe any methods used to tabulate or visually display results of individual studies and syntheses.
	13d	Describe any methods used to synthesise results and provide a rationale for the choice(s). If meta‐analysis was performed, describe the model(s), method(s) to identify the presence and extent of statistical heterogeneity and software package(s) used.
	13e	Describe any methods used to explore possible causes of heterogeneity among study results (e.g., subgroup analysis, meta‐regression).
	13f	Describe any sensitivity analyses conducted to assess robustness of the synthesised results.
Reporting bias assessment	14	Describe any methods used to assess risk of bias due to missing results in a synthesis (arising from reporting biases).
Certainty assessment	15	Describe any methods used to assess certainty (or confidence) in the body of evidence for an outcome.
Study selection	16a	Describe the results of the search and selection process, from the number of records identified in the search to the number of studies included in the review, ideally using a flow diagram.
	16b	Cite studies that might appear to meet the inclusion criteria, but which were excluded, and explain why they were excluded.
Study characteristics	17	Cite each included study and present its characteristics.
Risk of bias in studies	18	Present assessments of risk of bias for each included study.
Results of individual studies	19	For all outcomes, present, for each study: (a) summary statistics for each group (where appropriate) and (b) an effect estimate and its precision, ideally using structured tables or plots.
Results of syntheses	20a	For each synthesis, briefly summarise the characteristics and risk of bias among contributing studies.
	20b	Present results of all statistical syntheses conducted. If meta‐analysis was done, present for each the summary estimate and its precision and measures of statistical heterogeneity. If comparing groups, describe the direction of the effect.
	20c	Present results of all investigations of possible causes of heterogeneity among study results.
	20d	Present results of all sensitivity analyses conducted to assess the robustness of the synthesised results.
Reporting biases	21	Present assessments of risk of bias due to missing results (arising from reporting biases) for each synthesis assessed.
Certainty of evidence	22	Present assessments of certainty (or confidence) in the body of evidence for each outcome assessed.
Discussion	23a	Provide a general interpretation of the results in the context of other evidence.
	23b	Discuss any limitations of the evidence included in the review.
	23c	Discuss any limitations of the review processes used.
	23d	Discuss implications of the results for practice, policy and future research.
Registration and protocol	24a	Provide registration information for the review, including register name and registration number or state that the review was not registered.
	24b	Indicate where the review protocol can be accessed, or state that a protocol was not prepared.
	24c	Describe and explain any amendments to information provided at registration or in the protocol.
Support	25	Describe sources of financial or non‐financial support for the review, and the role of the funders or sponsors in the review.
Competing interests	26	Declare any competing interests of review authors.
Availability of data, code and other materials	27	Report which of the following are publicly available and where they can be found: template data collection forms; data extracted from included studies; data used for all analyses; analytic code; any other materials used in the review.
